# THE FLUID COMPLEXITY OF RESIDENTIAL CHOICES IN LATER LIFE

**DOI:** 10.1093/geroni/igz038.817

**Published:** 2019-11-08

**Authors:** Jaber F Gubrium

**Affiliations:** University of Missouri, Columbia, Missouri, United States

## Abstract

Serious attention to the fluid complexity of lived experience throws intriguing light on residential choices in later life. Fluidity comes in the form of time, which shifts in both novel and patterned ways as one ages. Decisions in time not only follow well-documented patterns, but interpersonal and intersectional considerations, both retrospectively and prospectively, can alter the essential meaning and consequences of ostensible patterns in the matter. Complexity emerges when the interconnected meanings of individual variables are taken into account. Working together, fluidity and complexity turn what is otherwise well-documented into what might be called forms of “assemblage” of accountability and decision-making in residential choices.

